# The Beneficial Effects of Combining Anti-Aβ Antibody NP106 and Curcumin Analog TML-6 on the Treatment of Alzheimer’s Disease in APP/PS1 Mice

**DOI:** 10.3390/ijms23010556

**Published:** 2022-01-05

**Authors:** Ih-Jen Su, Chia-Yu Hsu, Santai Shen, Po-Kuan Chao, John Tsu-An Hsu, Jung-Tsung Hsueh, Jia-Jun Liang, Ying-Ting Hsu, Feng-Shiun Shie

**Affiliations:** 1 Merry Life Biomedical Company, Ltd., 1F., No. 186, Daqiao 2nd St., Yongkang Dist., Tainan City 71048, Taiwan; suihjen0704@stust.edu.tw (I.-J.S.); rd03@tmlbio.com (C.-Y.H.); gary902202@nhri.edu.tw (J.-T.H.); 2 Center for Neuropsychiatric Research, National Health Research Institutes, Miaoli County 35053, Taiwan; Santai.Shen@antaimmu.com (S.S.); hubert@nhri.edu.tw (P.-K.C.); ljj5827@nhri.edu.tw (J.-J.L.); redff0422@nhri.edu.tw (Y.-T.H.); 3 Institute of Biotechnology and Pharmaceutical Research, National Health Research Institutes, Miaoli County 35053, Taiwan; tsuanhsu@nhri.edu.tw

**Keywords:** Alzheimer’s disease, combination treatment, anti-Aβ immunotherapy, microglial phagocytosis, inflammation, gut microbiota

## Abstract

Alzheimer’s disease (AD) is a progressive neurodegenerative disease with a multifactorial etiology. A multitarget treatment that modulates multifaceted biological functions might be more effective than a single-target approach. Here, the therapeutic efficacy of combination treatment using anti-Aβ antibody NP106 and curcumin analog TML-6 versus monotherapy was investigated in an APP/PS1 mouse model of AD. Our data demonstrate that both combination treatment and monotherapy attenuated brain Aβ and improved the nesting behavioral deficit to varying degrees. Importantly, the combination treatment group had the lowest Aβ levels, and insoluble forms of Aβ were reduced most effectively. The nesting performance of APP/PS1 mice receiving combination treatment was better than that of other APP/PS1 groups. Further findings indicate that enhanced microglial Aβ phagocytosis and lower levels of proinflammatory cytokines were concurrent with the aforementioned effects of NP106 in combination with TML-6. Intriguingly, combination treatment also normalized the gut microbiota of APP/PS1 mice to levels resembling the wild-type control. Taken together, combination treatment outperformed NP106 or TML-6 monotherapy in ameliorating Aβ pathology and the nesting behavioral deficit in APP/PS1 mice. The superior effect might result from a more potent modulation of microglial function, cerebral inflammation, and the gut microbiota. This innovative treatment paradigm confers a new avenue to develop more efficacious AD treatments.

## 1. Introduction

Alzheimer’s disease (AD) is the most common form of dementia, and manifestations of progressive memory loss and cognitive dysfunction are typical clinical characteristics [1,2]. While a cure is not available, amyloid β (Aβ), a major component of Aβ plaques, is an attractive therapeutic target for drug design, and many disease-modifying strategies aim to prevent Aβ production and/or enhance its clearance for AD therapy [3,4,5]. Microglia are the predominant immune cells in the brain, and are mainly responsible for Aβ clearance through phagocytosis. Although microglial functions may be independent of morphological changes [6], microglia transform from a ramified morphology in the healthy state, in which cells are highly branched and have multiple long processes, to an amoeboid/de-ramified morphology in the activated state. Overactivated microglia in the vicinity of Aβ plaques in AD brains are de-ramified during disease progression. They have enlarged cell bodies and a phagocytic phenotype, which facilitates Aβ clearance. However, chronic overactivation can lead to microglial frustration, and these frustrated microglia are dysfunctional amoeboid microglia that consequently become senescent and lose their Aβ phagocytic activity [7]. Moreover, chronic microglial overactivation exacerbates neuroinflammation, which has been thought to be involved in the progression of AD [8,9]. Thus, the restoration of normal microglial function holds great significance and may constitute a practicable therapeutic strategy for AD [10,11].

It has been recently recognized that the gut microbiota may influence brain functioning, while the gut microbiota composition is altered in AD and with aging [12,13]. Therefore, this gut–brain axis may contribute to the pathogenesis of AD. Although this bidirectional gut–brain axis is not fully understood in AD, increasing evidence from animal studies demonstrates that associations between the gut microbiota and AD pathology can be mediated by neural, endocrine, and immune signals [14]. The gut microbiota affect microglial functions and regulate their gene expression in response to pathological conditions [15,16]. While short-chain fatty acids (SCFAs), derived from bacterial metabolites and comprised of acetate, propionate, and butyrate, have been identified as signaling molecules, the mechanisms underlying the neuroactive effects of SCFAs on microglial function and neuro-immuno-endocrine regulation remain largely unknown [15,17]. Notably, a butyrate-producing genera was reduced in patients with AD [18], and treatments involving butyrate-producing bacteria were reported to be beneficial against AD-like pathology and were able to prevent behavioral deficits in mice [19]. 

In recent clinical trials, passive immunotherapy using anti-Aβ antibodies has shown the most promise for slowing down Aβ accumulation in patients with AD [20,21]. However, high doses of anti-Aβ antibody are limited by increased risks of amyloid-related imaging abnormalities, especially in patients with apolipoprotein E ε4 allele-associated AD [22]. Recently, aducanumab, marketed as Aduhelm by Biogen/Eisai, has been approved by the U.S. Food and Drug Administration for slowing disease progression in mild AD, although its efficacy in cognition improvement remains debatable [23,24]. The potential beneficial effects of aducanumab on delaying the progression of AD are mainly due to the antibody-induced microglia-mediated Aβ clearance. However, the Aβ disposal concept that can effectively cure AD is controversial. In fact, brain pathology has been proposed to occur up to 20 years before the onset of clinical manifestations of AD [25,26]. By the time AD is diagnosed, the disease has progressed too far to be managed. Severely compromised brain function due to chronic pathological insults makes AD treatment more difficult, and a multitarget, rather than one-target treatment strategy, is required for this complex disease [27,28]. A favorable combination of decreased microglia-mediated neuroinflammation and enhanced Aβ clearance has been proposed as a promising therapeutic paradigm [29].

We recently developed an anti-Aβ antibody NP106 [30], and TML-6 is a novel synthetic curcumin analog that possesses antiaging and anti-inflammatory properties through multiple mechanisms as reported elsewhere [31]. It has been shown to activate the antioxidant, nuclear factor erythroid 2-related factor 2, and reduce the phosphorylation of nuclear factor-kappa B and the mammalian target of rapamycin. In addition, it reduced the production and accumulation Aβ in vitro and in vivo. In this study, the efficacy of a combination of NP106 with TML-6 was compared to monotherapy in APP/PS1 mice, and the data show that combination treatment achieved favorable multitarget therapeutic effects and outperformed monotherapy using either drug.

## 2. Results

### 2.1. Combination Treatment Outperformed NP106 or TML-6 Monotherapy in Reducing Aβ Levels in the Brain of APP/PS1 Mice

**Figure 1:**
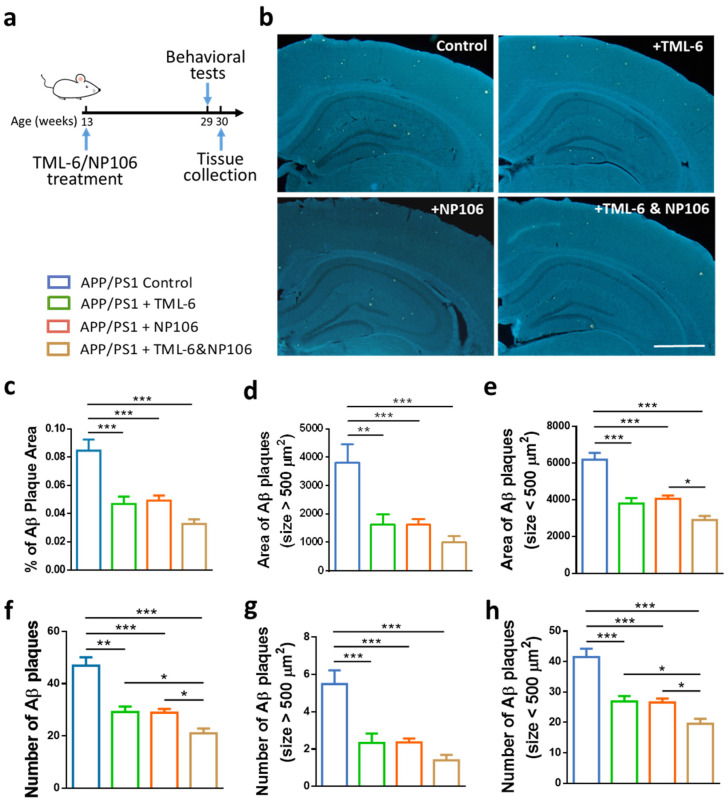
Treatment effects on the reduction of Aβ plaques in the brains of APP/PS1 mice. (**a**) Diagram of the study design. (**b**) Representative images of Amylo-glo-stained Aβ plaques in the brain of APP/PS1 mice after 17 weeks of treatment. (**c**) Quantification of the percentage (%) of Aβ plaque area. (**d**) The area of Aβ plaques larger than 500 μm^2^. (**e**) The area of Aβ plaques smaller than 500 μm^2^. (**f**) The number of Aβ plaques. (**g**) The number of Aβ plaques larger than 500 μm^2^. (**h**) The number of Aβ plaques smaller than 500 μm^2^. Data are shown as mean ± standard error of the mean per brain section, and one-way ANOVA with Tukey’s multiple comparison test was performed. * *p* < 0.05; ** *p* < 0.01; *** *p* < 0.001. The number of animals is 9, 10, 11, and 11 for the APP/PS1 control, APP/PS + TML-6, APP/PS1 + NP106, and APP/PS1 + TML-6 &NP106, respectively. Scale bar: 1000 μm.

**Figure 2:**
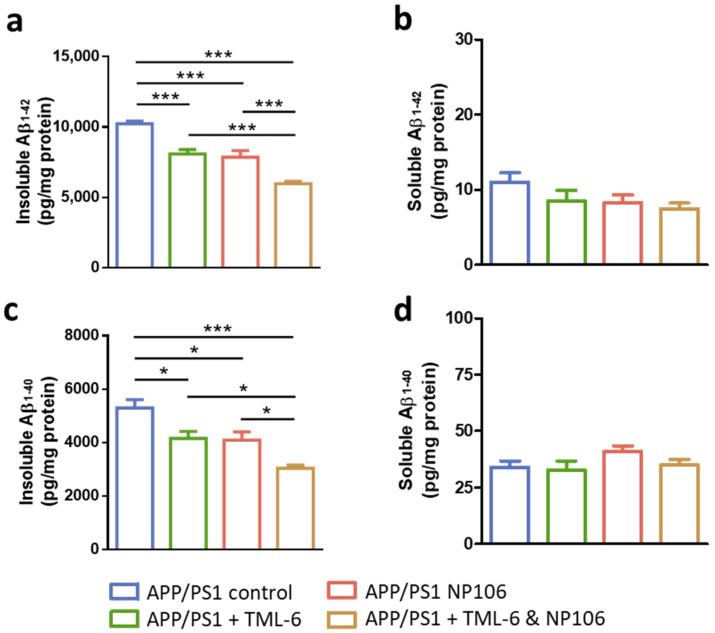
Insoluble and soluble forms of Aβ_1–42_ and Aβ_1–40_ in the brains of APP/PS1 mice. (**a**) Levels of insoluble Aβ_1–42_. (**b**) Levels of soluble Aβ_1–42_. (**c**) Levels of insoluble Aβ_1–40_. (**d**) Levels of soluble Aβ_1–40_. Data are shown as mean ± standard error of the mean per brain section, and one-way ANOVA with Tukey’s multiple comparison test was performed. * *p* < 0.05; *** *p* < 0.001. The number of animals is 9, 10, 11, and 11 for the APP/PS1 control, APP/PS1 + TML-6, APP/PS1 + NP106, and APP/PS1 + TML-6 & NP106, respectively.

APP/PS1 mice were subjected to 17-week treatments of NP106, TML-6, or combined NP106/TML-6 as illustrated in Figure 1a. Aβ deposition as measured by Amylo-Glo staining was apparent in both the cortex and hippocampus of the 30-week-old APP/PS1 mice (Figure 1b). Mice receiving NP106 or TML-6 monotherapy or combination treatment had lower levels of Aβ deposition than those in the APP/PS1 control group. Quantification of Aβ deposition indicated that the percentage area of Aβ plaques in the brain was significantly reduced by both combination treatment and monotherapy, while Aβ deposition for mice in the combination treatment group was slightly lower than that for mice with monotherapy (Figure 1c). A similar reduction pattern was also found in Aβ plaques larger than 500 μm^2^ (Figure 1d), Aβ plaques smaller than 500 μm^2^ (Figure 1e), and the number of Aβ plaques (Figure 1f–h). Combination treatment seems to be better than monotherapy in reducing the total number of Aβ plaques and the number of smaller Aβ plaques. Furthermore, insoluble forms of Aβ_1–42_ (Figure 2a) and Aβ_1–40_ (Figure 2c) in the brain were significantly reduced by both combination treatment and monotherapy, while combination treatment led to a further significant reduction. However, no significant difference was found among all groups for levels of the soluble forms of Aβ_1–42_ (Figure 2b) and Aβ_1–40_ (Figure 2d).

### 2.2. Effects of Combination Treatment on the Nesting Behavioral Test

**Figure 3:**
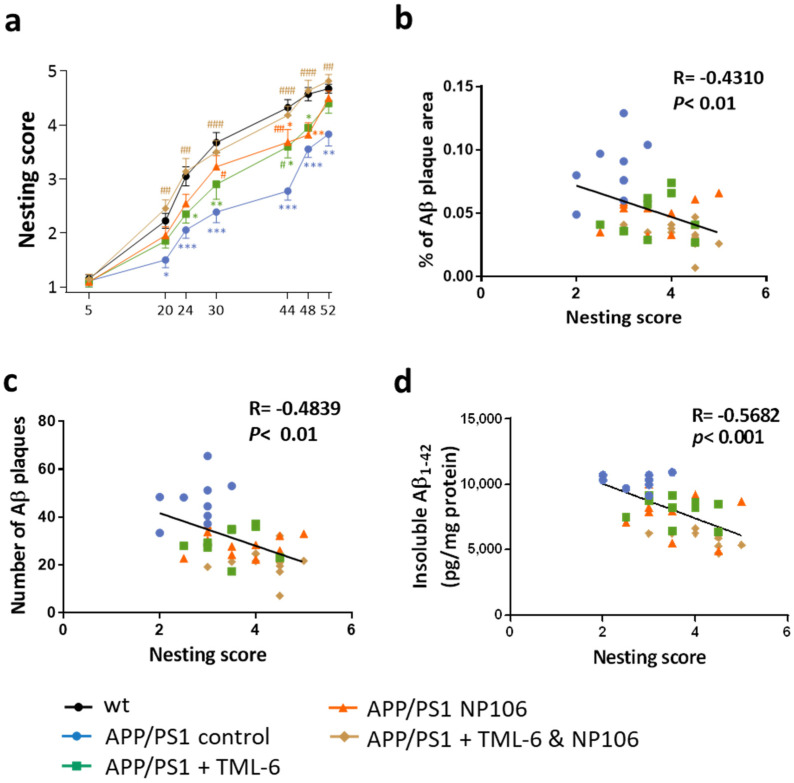
Nesting behavioral tests and the correlations between nesting scores and Aβ pathology. (**a**) Nesting scores at indicated time points. Two-way ANOVA with Tukey’s multiple comparison test was performed. * *p* < 0.05; ** *p* < 0.01; *** *p* < 0.001, comparison between APP/PS1 control mice (n = 9) and the wt control mice (n = 20); * *p* < 0.05, ** *p* < 0.01, comparison between APP/PS1 mice receiving TML-6 (n = 10) and the wt control mice; * *p* < 0.05, ** *p* < 0.01, comparison between APP/PS1 mice receiving NP106 (n = 11) and the wt control mice; # *p* < 0.05, comparison between APP/PS1 mice receiving TML-6 and APP/PS1 control mice; # *p* < 0.05, ## *p* < 0.01, comparison between APP/PS1 mice receiving NP106 and APP/PS1 control mice; ## *p* < 0.01, ### *p* < 0.001, comparison between APP/PS1 mice receiving combination treatment (n = 11) and APP/PS1 control mice. (**b**) The correlations between the nesting scores and the percentage (%) of Aβ plaque area per brain section. (**c**) The correlations between the nesting scores and the number of Aβ plaques per brain section. (**d**) The correlations between the nesting scores and the levels of insoluble Aβ_1–42_ in the brain.

As expected, APP/PS1 control mice had lower nesting scores than those in the wild-type (wt) control group (Figure 3a), indicating dysfunctional brain neuronal networking in APP/PS1 mice. In line with the reduction effect on Aβ in the brain, NP106 or TML-6 monotherapy resulted in a significant improvement in the nesting behavioral test at the indicated time points as compared to the APP/PS1 control, while the nesting scores of mice with monotherapy were also significantly different from those of the wt control. The nesting scores of mice receiving combination treatment were closer to or even higher than those of the wt control throughout the test, and were statistically comparable to those of the wt control, but significantly different from those of the APP/PS1 control and APP/PS1 mice with monotherapy. These data suggest that combination treatment is better than monotherapy for reversing the abnormal nesting ability of APP/PS1 mice. Since the nesting scores and the brain Aβ levels could be modulated by the treatments, the relationship between these two disease indications in APP/PS1 mice was examined. Correlation analyses were analyzed using the final nesting scores at 52 h and the measurements of brain Aβ. As shown in Figure 3b–d, nesting scores were negatively correlated with insoluble Aβ_1–42_ levels (r = −0.5682, *p* < 0.001), the percentage of Aβ plaques (r = −0.431, *p* < 0.01), and the number of Aβ plaques (r = −0.4783, *p* < 0.01). However, nesting scores were not significantly correlated with soluble Aβ_1–42_ (r = −0.2985, *p* = 0.058), insoluble Aβ_1–40_ (r = −0.1763, *p* = 0.2702), and soluble Aβ_1–40_ (r = 0.03743, *p* = 0.8163). These data suggest that reducing Aβ plaques and insoluble Aβ_1–42_ levels, but not insoluble Aβ_1–40_, which in the brain is associated with the improvement of impaired brain function, can be better achieved by combination treatment in APP/PS1 mice.

### 2.3. Effects of Combination Treatment on Microglial Aβ Phagocytosis and the Morphological Changes of Reactive Microglia

**Figure 4:**
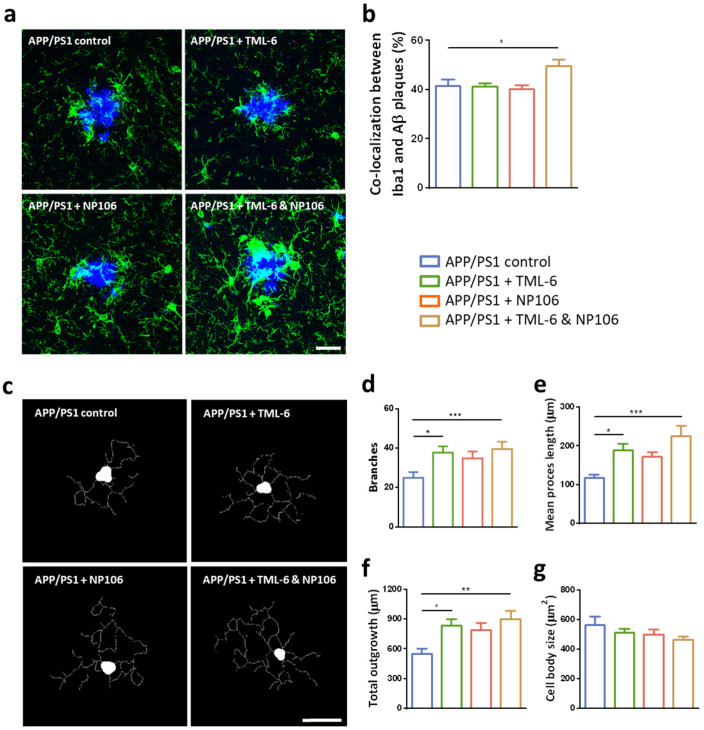
The effects of combination treatment on Aβ phagocytosis and morphological changes in microglia. (**a**) Representative confocal images showing colocalization of the immunoreactivity of Iba1 (green) and Aβ plaques (blue) in the brain of APP/PS1 mice without or with treatments. (**b**) Quantification of colocalization of Iba1 immunoreactivity within Aβ plaques (in %). (**c**) Representative skeletonized images from morphological analyses of the microglia. Quantification of morphological analyses indicating the number of branches (**d**), mean process length (**e**), total outgrowth (**f**), and cell body size (**g**) of the microglia. One-way ANOVA with Dunnett’s multiple comparison test was performed. Data are shown as mean ± standard error of the mean. * *p* < 0.05; ** *p* < 0.01; *** *p* < 0.001. The number of animals is 9, 10, 11, and 11 for the APP/PS1 control, APP/PS1 + TML-6, APP/PS1 + NP106, and APP/PS1 + TML-6 & NP106, respectively. Scale bars: 25 μm.

To understand the possible mechanism underlying the aforementioned effects of combination treatment, microglial function was explored. Microglia are the primary immune cells responsible for Aβ clearance in the brain, and colocalization of the immunoreactivity of Iba1, a microglial marker, within Aβ plaques has been used to indicate microglial Aβ phagocytosis. Representative confocal images are shown in Figure 4a with quantification in Figure 4b. Data shows that there is no change in the percentage of colocalization between microglia and Aβ plaques after the treatments of either NP106 or TML-6 monotherapy. However, it was significantly elevated in the combination treatment group, suggesting that microglial Aβ phagocytosis was significantly enhanced by the combination of NP106 and TML-6, but not by monotherapy. To investigate whether the enhanced microglial Aβ phagocytosis was concomitant with the morphological changes, confocal images of brain sections were subjected to a morphological analysis using MetaMorph software. Representative skeletonized images from APP/PS1 mice without or with treatments are presented in Figure 4c. Quantitative data indicate that the number of branches (Figure 4d), mean process length (Figure 4e), and total outgrowth (Figure 4f) of the microglia were significantly increased by both TML-6 monotherapy and combination treatment, while a slight increase was found in the NP106 group. The combination treatment group seems to have the smallest size of cell body in all groups, but no significance was found (Figure 4g). These data suggest that combination treatment promoted microglial Aβ phagocytosis concurrently with a significant morphological transformation into a characteristic ramified morphology with multiple long branches.

### 2.4. Anti-Inflammatory Effects of Combination Treatment on the Reduction of Cerebral Proinflammatory Cytokines

**Figure 5:**
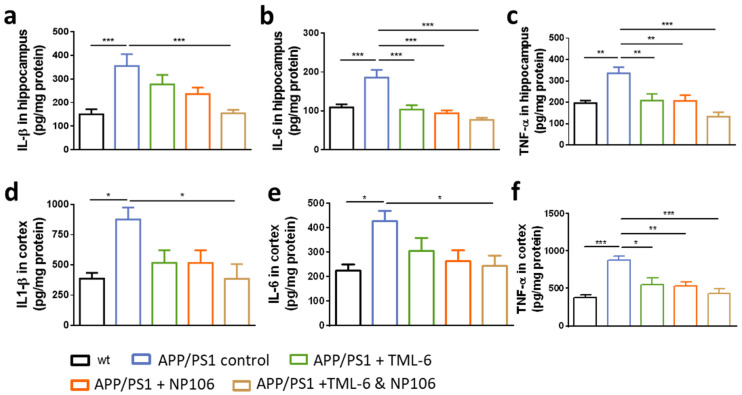
The anti-inflammatory effects of combination treatment on the levels of proinflammatory cytokines. Levels of IL1β (**a**,**d**), IL-6 (**b**,**e**) and TNFα (**c**,**f**) in hippocampus or cortex as measured by ELISA are presented. One-way ANOVA with Tukey’s multiple comparison test was performed. Data are shown as mean ± standard error of the mean. * *p* < 0.05; ** *p* < 0.01; *** *p* < 0.001. The number of animals is 9, 10, 11, and 11 for the APP/PS1 control, APP/PS1+TML-6, APP/PS1+NP106, and APP/PS1+TML-6&NP106, respectively.

Next, the levels of proinflammatory cytokines, including IL-1β, IL-6 and TNFα, in the brain were examined. Results show that IL-1β levels were higher in APP/PS1 control mice than those in the wt control. While combination treatment significantly reduced IL-1β in both the hippocampus (Figure 5a) and the cortex (Figure 5d), levels of IL-1β were slightly reduced by either TML-6 or NP106 monotherapy, albeit not significantly. Similarly, IL-6 (Figure 5b,e) and TNFα (Figure 5c,f) were significantly reduced by combination treatment. TNFα levels (Figure 5c,f) were also reduced by TML-6 and NP106 monotherapy, while a significant reduction of IL-6 by monotherapy was found in the hippocampus (Figure 5b), but not in the cortex (Figure 5e). 

### 2.5. Combination Treatment Normalized Aberrant Bacterial Communities in APP/PS1 Mice to wt Levels

**Figure 6:**
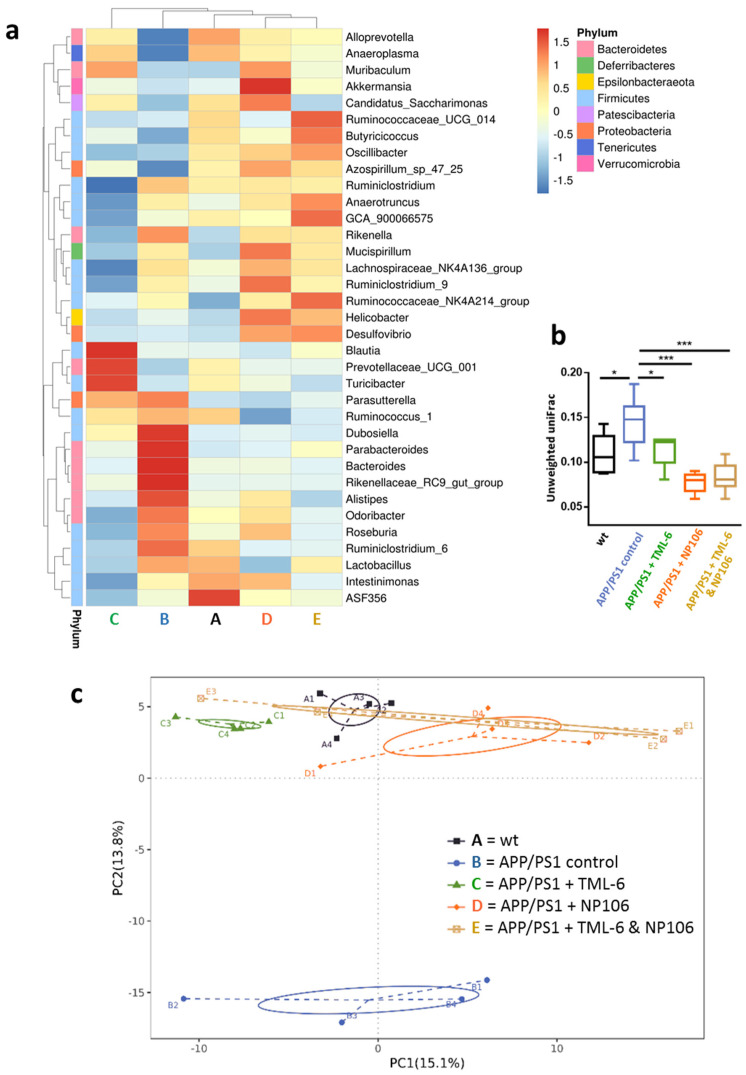
Alterations of the bacterial communities in APP/PS1 mice by all the treatments. (**a**) A cluster heat map of the 35 most abundant bacterial genera is presented. The abscissa of the cluster heat map denotes the five study groups, and the right ordinate denotes bacterial genera. The left ordinate shows the cluster tree of the genera, and the phylum is indicated using colored blocks. The corresponding value in the legend of the heat map is the z-score obtained by normalizing the abundance of each genus in all groups. (**b**) The effects of all the treatments on the structural differences in the bacterial communities of all groups by Unweighted uniFrac analyses. (**c**) Principal coordinate analysis of gut bacteria indicates that the bacterial communities of APP/PS1 control mice are different from those of the wt control and APP/PS1 mice receiving all the treatments, while those of APP/PS1 mice treated with combination treatment are more similar to those of the wt control than to those of other treatment groups. One-way analysis of variance with Tukey’s multiple comparison test was performed. * *p* < 0.05; *** *p* < 0.001. n = 4 per group.

Given that the gut–brain axis has gained more and more attention due to its important role in the pathogenesis of AD, the gut microbiota was investigated in this study. Fecal analyses were applied to explore whether modulation of the gut microbiota explains the beneficial effects of combination treatment on the reduction of Aβ levels and the improvement of the nesting behavioral deficit in APP/PS1 mice. A cluster heat map of the 35 most abundant genera is presented in Figure 6a to show the bacterial communities of the wt and APP/PS1 mice with and without treatment. We found that the gut microbiome of APP/PS1 mice receiving NP106 monotherapy or combination treatment tended to be more similar to the wt control than to the APP/PS1 control. Compared to the wt control, APP/PS1 control mice had higher levels (z-score ≥ 1.5, in red color) of Dubosiella, Parabacteroides, Bacteroides, and Rikenellaceae RC9 gut group bacteria and lower levels (z-score ≤ 1.5, in blue color) of Alloprevotella and Anaeroplasma bacteria. These changes were reversed by all treatments. Unweighted uniFrac analysis also showed that the significant structural differences in the bacterial communities between the APP/PS1 and wt control were abolished by all treatments, and the bacterial communities of APP/PS1 mice receiving TML-6 (*p* < 0.05), NP106 monotherapy (*p* < 0.001) or combination treatment (*p* < 0.001) were significantly different from those of APP/PS1 control mice (Figure 6b). A principal coordinate analysis (Figure 6c) further indicated that the bacterial communities of the APP/PS1 control mice were different from those of the wt control and the APP/PS1 mice receiving all treatments, and that the bacterial communities of APP/PS1 mice receiving combination treatment were more similar to those of the wt control than those of the other treatment groups.

These effects of combination treatment on bacterial communities were confirmed by analyses of among-group differences, such as an analysis of group similarities (ANOSIM), multi-response permutation procedures (MRPP) and a nonparametric multivariate analysis of variance (ADONIS). As shown in Table 1, mice with monotherapy were significantly different from APP/PS1 control mice by three different analyses, but all of them were also significantly different from the wt control mice, except for the NP106-treated mice in comparison with the wt control on ADONIS (*p* = 0.057). Importantly, APP/PS1 mice receiving combination treatment were significantly different from APP/PS1 control mice and were comparable to the wt control mice on ANOSIM (*p* = 0.124), MRPP (*p* = 0.103), and ADONIS (*p* = 0.140). These results suggest that combination treatment is more effective than monotherapy to normalize the aberrant bacterial communities in APP/PS1 mice to levels resembling the wild-type control. 

**Figure 7:**
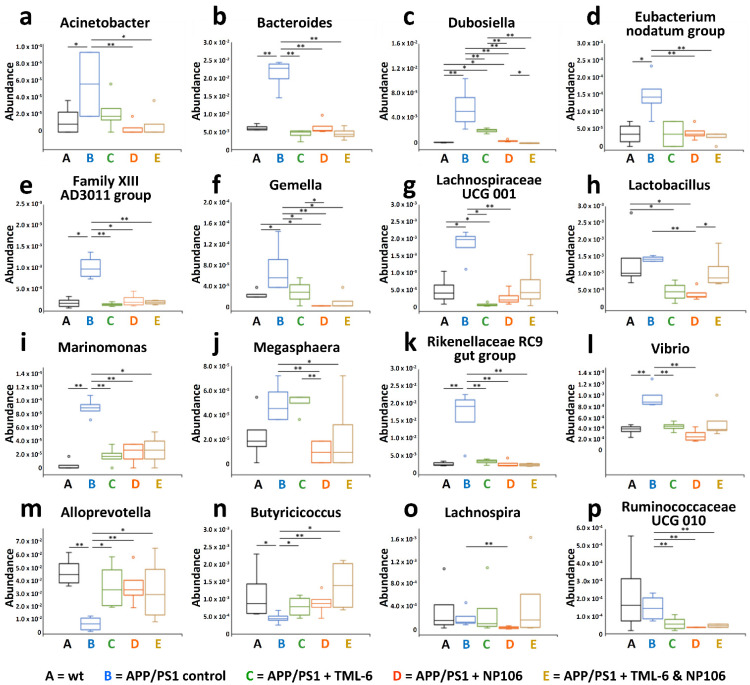
The alterations of bacterial communities in APP/PS1 mice by all the treatments. Among sixteen bacterial genera identified in this study, ten bacterial genera, namely Acinetobacter (**a**), Bacteroides (**b**), Dubosiella (**c**), Eubacterium nodatum group (**d**), Family XIII AD3011 group (**e**), Gemella (**f**), Lachnospiraceae UCG 001 (**g**), Marinomonas (**i**), Rikenellaceae RC9 gut group (**k**), and Vibrio (**l**), had a significantly increased abundance in APP/PS1 control mice. In contrast, two bacterial genera, namely Alloprevotella (**m**) and Butyricicoccus (**n**), had a significantly reduced abundance in APP/PS1 control mice. Lactobacillus (**h**), Megasphaera (**j**), Lachnospira (**o**), and Ruminococcaceae UCG 010 (**p**) had no significantly changed abundances in the APP/PS1 control mice. A Kruskal–Wallis rank sum test was performed. The genus names are indicated in each boxplot, and outliers (circle) outside the interquartile range are indicated. n = 4 per group. * *p* < 0.05; ** *p* < 0.01.

From the microbiota analyses, 16 bacterial genera (Figure 7a–p) with significant alterations in abundance were identified as described in Methods. Compared with the wt control mice, 10 out of the 16 genera were significantly increased in APP/PS1 control mice, while two bacterial genera were significantly reduced. Among ten increased bacterial genera (Acinetobacter, Bacteroides, Dubosiella, Eubacterium nodatum group, Family XIII AD3011 group, Gemella, Lachnospiraceae UCG 001, Marinomonas, Rikenellaceae RC9 gut group, and Vibrio), six of them (Figure 7b,c,e,f,i,k) were significantly reversed to lower levels by all the treatments. Intriguingly, APP/PS1 mice with combination treatment had the lowest abundance of the genera Dubosiella (Figure 7c) compared to all the APP/PS1 mice without or with monotherapy. On the other hand, the abundances of two bacterial genera (Alloprevotella and Butyricicoccus) were reduced in APP/PS1 control mice, and they were increased by all the treatments (Figure 7m,n).

### 2.6. Associations of Abundances of Bacterial Genera with the Severity of Cerebral Aβ Pathology and Nesting Behavioral Abnormality

We then examined whether the abundance of the identified bacterial genera was associated with the nesting scores. Further correlation analysis on the 16 bacterial genera showed that the abundances of eight bacterial genera were highly correlated with nesting performance (Table 2). Among them, the abundances of seven bacterial genera, namely Acinetobacter (*p* < 0.05), Bacteroides (*p* < 0.05), Dubosiella (*p* < 0.001), Eubacterium nodatum group (*p* < 0.001), Family XIII AD3011 group (*p* < 0.05), Marinomonas (*p* < 0.01), and Rikenellaceae RC9 gut group (*p* < 0.001), were increased in APP/PS1 control mice as compared to the wt control mice, and were negatively correlated with nesting scores. In contrast, the abundance of Butyricicoccus (*p* < 0.01) was decreased in APP/PS1 control mice, and it was positively correlated with nesting scores. Next, correlations of the abundance of the 16 bacterial genera with the area of Aβ plaques, number of Aβ plaques, area of large Aβ plaques (size > 500 μm^2^), and area of small Aβ plaques (size < 500 μm^2^) were studied. As shown in Table 2, the abundances of seven bacterial genera (Bacteroides, Dubosiella, Eubacterium nodatum group, Family XIII AD3011 group, Gemella, Marinomonas, and Rikenellaceae RC9 gut group) were positively correlated with Aβ pathology. Butyricicoccus, which had a decreased abundance in APP/PS1 control mice, was negatively correlated with the area of Aβ plaques, the number of Aβ plaques, and with small Aβ plaques (*p* < 0.05). In summary, these findings indicate that six bacterial genera (Bacteroides, Dubosiella, Eubacterium nodatum group, Family XIII AD3011 group, Marinomonas, and Rikenellaceae RC9 gut group, in a blue color) had increased abundances in APP/PS1 control mice as compared to the wt control mice and were positively and negatively correlated with Aβ pathology and nesting scores, respectively. The abundance of Butyricicoccus (in an orange color) in APP/PS1 mice was negatively and positively correlated with Aβ pathology and nesting scores, respectively. Together, these data indicate that the abundance of the aforementioned bacterial genera are highly correlated with the disease state of APP/PS1 mice, and all of these can be reversed by combination treatment and NP106, and partly by TML-6.

## 3. Discussion

Combination treatment with NP106 and TML-6 reduced Aβ pathology and improved the nesting behavioral deficit in APP/PS1 mice, and these effects were superior to those of NP106 or TML-6 monotherapy. Our findings echo the speculation that combination treatment, having multifaceted biological functions, can be more effective than monotherapy. Whether the beneficial effect of combination treatment is still valid for human beings and whether it is applicable for other anti-Aβ antibody drugs requires further investigation.

Here, we found that the beneficial effects of combination treatment on counteracting AD-like pathology in APP/PS1 mice were concurrent with its anti-inflammatory effects and microglial transformation into functional microglia, leading to enhanced microglial Aβ clearance. To the best of our knowledge, this is the first study to report the beneficial effects of combination treatment on microglial Aβ phagocytosis and on the morphological changes from a de-ramified/overactivated state to a ramified state. Since microglia are the primary immune cells responsible for Aβ phagocytosis, chronic microglial overactivation can cause microglial senescence and consequently they lose their function, leading to an accumulation of Aβ. In the vicinity of cerebral Aβ plaques in aging APP/PS1 mice, overactivated microglia become amoeboid or de-ramified, with enlarged cell bodies, fewer branches, and shorter processes. Our data show that microglia were rejuvenated by the combination treatment of NP106 and TML-6, and their ramified morphology resembled that of resting microglia in a healthy brain. These morphological changes toward a healthy morphology following combination treatment were in line with increased Aβ phagocytosis and a robust reduction of Aβ pathology. In addition, the anti-inflammatory effects of combination treatment exerting a downregulation of proinflammatory cytokines may create a favorable microenvironment to prevent microglial senescence, while facilitating the recovery of microglial functions. Therefore, revealing the mechanisms underlying the functional recovery and morphological transformation from dysfunctional microglia into rejuvenated ones may help us understand the pathogenesis of AD and fine-tune the implementation of combination treatment to combat AD. 

For the nesting behavior test, it has been used to evaluate executive functions and is considered a measurement of nonmemory/nonlearned behavior in animals, while nesting deficits can be associated with functional damage in the hippocampus [32,33,34,35,36,37]. Our findings demonstrate that nesting scores are highly sensitive and indicate improvements in both the cerebral Aβ pathology and gut microbiota in APP/PS1 mice. Therefore, the nesting behavioral test may be a valuable, convenient, and a sensitive tool for the evaluation of AD-like pathology in AD animal models.

In our study, *Bacteroides* of the family *Bacteroidaceae* was found to be the most abundant bacterial genus, and its abundance was significantly increased in APP/PS1 mice compared to the wt control. Furthermore, the abundances of *Bacteroides* and five other genera were positively associated with the severity of Aβ pathology and negatively associated with the nesting performance. These findings are consistent with reports of an increased abundance of *Bacteroides* in patients with AD or mild cognitive impairment. This microbiome has been considered as proinflammatory and may exacerbate AD pathology [13,38]. However, contrasting findings from some other studies reported that the abundance of *Bacteroides* was reduced in patients with AD and that *Bacteroides* can be a health-promoting microbiome [39,40,41]. 

On the other hand, we found that the abundance of the genus *Butyricicoccus* of the family *Ruminococcaceae,* which was associated with a better nesting performance and a reduced Aβ pathology, also seems to be the highest in the mice treated with combination treatment. Indeed, literature has shown that the abundance of *Butyricicoccus* in patients with AD and other inflammatory diseases was reduced [18,42]. *Butyricicoccus* is a genus of butyrate-producing bacteria. Butyrate can enhance the anti-inflammatory activity of peroxisome proliferator-activated receptor γ [43], and it is an important agent for promoting gut and brain health through gut–brain communication [17,44]. The effects of combination treatment on normalizing the gut microbiota of APP/PS1 mice to a level resembling the wt control highlight the role of the gut–brain axis in the progression of AD. Emerging evidence suggests that the bidirectional gut–brain axis plays an important role in the progression of AD and in gut health. These data suggest that the therapeutic effects of combining NP106 and TM-6 may be involved in the modulation of the gut–brain axis. However, our current study could not answer whether the potential beneficial effects on gut dysbiosis are through direct effects on the gut, indirect impacts from the improvements in AD pathology in the brain, or both. The mechanism through which combination treatment normalizes the gut microbiota merits further investigation.

In conclusion, combination treatment was more effective than TML-6 or NP106 monotherapy to reduce Aβ and reverse the nesting behavioral deficit in APP/PS1 mice. The underlying mechanism of these beneficial effects, which may be involved in enhancing microglial Aβ phagocytosis, reducing the levels of proinflammatory cytokines, and modulating the gut microbiota, merits further investigation. These findings are valuable for the development of an improved treatment regimen for AD. The main limitations of such a pharmacological approach in the treatment of AD might include the adverse side effects of the drug as well as the low drug concentration across the blood brain barrier in the brain. Increasing attention has been paid to some nonpharmacological interventions, such as noninvasive brain stimulation (NIBS), as potential alternatives to treat AD-related cognitive impairment [45,46,47,48,49,50]. In fact, NIBS techniques, such as transcranial electrical stimulation (tES) or repetitive transcranial magnetic stimulation (TMS), have been investigated for AD therapy [51,52], and perhaps, in the future, these novel treatment techniques could be applied to facilitate the effectiveness of pharmacological regimens.

## 4. Materials and Methods

### 4.1. Animals

For this study, we used 13-week-old APP/PS1 (B6.Cg[APPswe,PSEN1dE9]85Dbo/J) transgenic mice engineered to develop AD-like pathology and their wt littermates. The animals were housed under controlled temperature (24 ± 1 °C) and humidity (55–65%) conditions with a 12:12 h (07:00–19:00) light–dark cycle. Experiments were performed as approved by the Institutional Animal Care and Use Committees of the National Health Research Institutes (NHRI-IACUC-107071-M2-A). 

### 4.2. Preparation of TML-6 Chow and Estimate of the Daily Uptake of TML-6

To avoid the potential stresses associated with daily oral gavage, TML-6 was given to mice through a dietary supplement. Regular rodent chow (Laboratory Rodent Diet 5001) supplemented with TML-6 at 100 mg/kg rodent chow was provided by Merry Life Biomedical Company, Ltd., Tainan City, Taiwan. The content of TML-6 in rodent chow was analyzed using liquid chromatography–tandem mass spectrometry (LC–MS/MS) at the Development Center for Biotechnology, Taiwan, and the amount of TML-6 in rodent chow was approximately 0.795 mg/g chow. The chow was stored at 4 °C and was added to the mouse cages twice a week for ad libitum feeding. The daily uptake of TML-6 by APP/PS1 mice was estimated based on the average consumption of rodent chow supplemented with TML-6 over a period of eight days. Three APP/PS1 mice were individually housed in metabolic cages with an apparatus for estimating the daily food intake. The TML-6 uptake per kg body weight per day was calculated by multiplying the daily consumption of TML-6-supplemented chow by the amount of TML-6 in the chow and dividing it by the body weight of the mouse. For each mouse with a body weight of 0.03 kg that consumed 4.2 g of TML-6-supplemented chow daily, the average daily consumption of TML-6 was estimated to be 111.3 mg/kg/d.

### 4.3. Treatment Regimens

NP106 has been cloned into a pcDNA3.4 expression plasmid and was produced in an Expi-CHOTM Expression system followed by protein G purification. To study the effects of combination treatment, 13-week-old APP/PS1 mice (early/prodromal stage of AD) were randomly assigned into four groups and underwent treatment for 17 weeks. Their wt littermates (n = 20) were included as a wt control group and underwent behavioral tests at the age of 29 weeks (1 week before sacrifice). Due to the spontaneous death of some APP/PS1 mice, pathological and behavioral examinations were conducted in 9, 10, 11, and 11 mice allocated to the APP/PS1 control mice, TML-6 monotherapy, NP106 monotherapy, and combination therapy groups, respectively. APP/PS1 mice received regular rodent chow and weekly intraperitoneal (ip) injections of 0.9% saline (APP/PS1 control group), rodent chow supplemented with TML-6 and weekly ip injections of 0.9% saline (TML-6 group), regular rodent chow and weekly ip injections of 3 mg/kg body weight NP106 (NP106 group), or rodent chow supplemented with TML-6 and weekly ip injections of 3 mg/kg body weight NP106 (NP106/TML-6 combination group). The body weight of all the mice was recorded weekly, which was comparable in all groups during the entire study period. Samples were harvested and were examined for experimental measurements after 17 weeks of treatment, at the age of 30 weeks. 

### 4.4. Pathological and Morphological Examination Using Confocal Microscopy

Brain sections from the right hemisphere of the brain were fixed in 4% paraformaldehyde followed by cryoprotection with 30% sucrose in 1× phosphate-buffered saline (PBS). Floating sections of 30 µm thickness were stored in 1× PBS with 0.05% sodium azide at 4 °C until use. Amylo-Glo RTD™ (TR-400-AG, Biosensis, Thebarton, Australia) was used per the manufacturer’s instructions to detect Aβ plaques in the brain sections. The sections were mounted on slides covered with mounting medium (Vectashield H-1000, Vector Laboratories, Burlingame, CA, USA). Images were acquired using a Leica confocal microscopy imaging system (Leica Microsystems, Wetzlar, Germany). The number of Aβ deposits and fluorescent area with Aβ deposits were normalized to the total area of the section using MetaMorph imaging software (Version 7.8.13, Molecular Devices, San Jose, CA, USA). Aβ deposits larger or smaller than 500 μm^2^ were analyzed. For colocalization, sections were stained with Amylo-Glo, followed by a three hour-incubation with microglia-specific antibodies (Iba1, Abcam, Cambridge, CB2 0AX, UK) at 1:100 dilution. Secondary antibodies were conjugated using Alexa Fluor 488 (Invitrogen), and the sections mounted on the slide were analyzed using a Leica confocal microscopy imaging system. Colocalization was quantified using confocal images from five brain sections per animal, and data obtained from the MetaMorph imaging software were normalized to the cortical and hippocampal regions of the brain. To analyze the microglial morphology, eight Iba1-positive cells near Aβ plaques were chosen from five brain sections per animal, and confocal images were analyzed using MetaMorph outgrowth analysis software. The measurements, including the number of branches, mean process length, total outgrowth, and size of cell body, are presented. 

### 4.5. Measurement of Levels of Aβ and Proinflammatory Cytokines Using ELISA

The frozen mouse brains from the left hemisphere of the brain were homogenized in 1× PBS (20% homogenate) containing a protease inhibitor cocktail (Sigma, St. Louis, MO, USA). To measure the levels of the soluble and insoluble forms of Aβ, 100 μL of 1% sodium dodecyl sulphate (SDS, Sigma) in PBS was added to 100 μL of homogenate, followed by ultracentrifugation at 175,000× *g* for 20 min at 4 °C. The resulting supernatant was the SDS-soluble Aβ. The pellet was then dissolved in 3 M guanidine HCl (Sigma) for 4 h at 4 °C, followed by ultracentrifugation at 175,000× *g* for 20 min at 4 °C. The resulting supernatant was the insoluble form of Aβ. ELISA kits for Aβ_1–40_ and Aβ_1–42_ (Invitrogen-Thermo Fisher Scientific, Carlsbad, CA, USA) were used according to the manufacturer’s instructions. Levels of IL-1β, IL-6 and TNFα in brain homogenates were measured by ELISA kits (R&D Systems, Minneapolis, MN USA) according to the manufacturer’s instructions. Results were analyzed using an ELISA reader (SpectraMaxM2, Molecular Devices) at a wavelength of 450 nm. Values were expressed as pg/mg protein. 

### 4.6. Nesting Behavioral Test

Two weeks before the pathological examinations, the mice were individually housed for 5 h before the nesting test. A Nestlet pressed-cotton square (Ancare, Bellmore, NY, USA) was placed in each cage 1 h before the beginning of the dark cycle. Photographs of the Nestlet were taken at specific time points, and the test lasted 52 h. Nest construction was graded on a five-point scale by two persons who were blinded to the treatments, as described in a previous study [53]. A score of 1 indicated that the Nestlet was >90% intact, whereas a score of 5 indicated that it was <10% intact and a nest with an obvious crater had been constructed.

### 4.7. Gut Microbiota Analysis Using 16S rDNA Sequencing

Fecal bacterial 16S rDNA was extracted from randomly selected animals (n = 4 per group). Fecal samples (0.2–0.3 g per mouse) were collected from the colons of the mice and immediately stored at −80 °C until use. DNA extraction and gut microbiota analysis were performed by Biotools Microbiome Research Center Co., Ltd., Taiwan. Briefly, DNA was extracted using the QIAamp Fast DNA Stool Kit (QIAGEN, Hilden, Germany) according to the manufacturer’s instructions. To analyze the phylogenetic composition of the gut microbiota, the V3–V4 region of the 16S rDNA gene was amplified, and 16S amplicon sequencing was performed using the Illumina MiSeq 2500 platform (San Diego, CA, USA). Operational taxonomic unit (OTU) clustering and unweighted UniFrac analysis were performed to compute a beta diversity distance matrix using Quantitative Insights Into Microbial Ecology version 1.9.0. Principal component analysis, partial least squares discriminant analysis, and a heatmap of redundancy analysis-identified key OTUs were used to analyze the beta diversity. The characteristics of the gut microbiota were analyzed using linear discriminant analysis (LDA) effect size (LEfSe) for biomarker discovery. LEfSe detected features with significant differences using the Kruskal–Wallis rank sum test and applied LDA to evaluate the effect size for each feature. Sixteen bacterial genera with significant alterations in abundance, as determined on metagenomeSeq analysis using a q significance that was normalized from the *p* significance, were identified. Data with a *p* significance were presented to show the differential abundance of bacterial genera.

### 4.8. Statistical Analyses

The statistical analyses of the gut microbiota data were described above, and Version 5.0 of Prism software (Graph Pad Software, Inc., La Jolla, CA, USA) was used for this study. Comparisons among groups were performed using the one-way or two-way analysis of variance (ANOVA) followed by Tukey’s multiple comparison post-hoc tests where applicable, and Dunnett’s post-hoc tests were used for comparisons with the control group. Pearson and Spearman correlations were computed where appropriate. The level of significance was set at *p* < 0.05.

## 5. Patents

The National Health Research Institutes hold the intellectual property of NP106, a novel anti-Aβ antibody protected by a United States patent (US 11,084,873B2) and Republic of China patent (#I721440), and Merry Life Biomedical Company holds the intellectual property of TML-6.

## Figures and Tables

**Table 1:** Analyses of among-group differences for the bacterial communities. ANOSIM, analysis of group similarities; MRPP, multi-response permutation procedures; ADONIS, nonparametric multivariate analysis of variance; A, the wt control; B, APP/PS1 control; C, TML-6 monotherapy; D, NP106 monotherapy; E, combination treatment. n = 4 per group. * *p* < 0.05.

	Anosim		MRPP		Adonis	
Group	R	*p* Value	Expected δ	*p* Value	R^2^	*p* Value
**A vs. B**	1	0.028 *	0.42	0.041 *	0.53	0.026 *
**A vs. C**	0.875	0.026 *	0.34	0.024 *	0.36	0.028 *
**A vs. D**	0.490	0.049 *	0.36	0.034 *	0.27	0.057
**A vs. E**	0.281	0.124	0.35	0.103	0.22	0.140
**B vs. C**	1	0.029 *	0.44	0.030 *	0.62	0.028 *
**B vs. D**	0.906	0.031 *	0.43	0.035 *	0.48	0.028 *
**B vs. E**	0.938	0.031 *	0.44	0.037 *	0.49	0.028 *
**C vs. D**	0.865	0.026 *	0.39	0.022 *	0.43	0.026 *
**C vs. E**	0.438	0.024 *	0.37	0.027 *	0.36	0.030 *
**D vs. E**	0.104	0.194	0.36	0.224	0.18	0.223

**Table 2:** The correlations between the abundance of sixteen bacterial genera and the nesting scores or Aβ pathology. Among sixteen bacterial genera, the abundances of six bacterial genera (highlighted in blue), which had an increased abundancy in APP/PS1 control mice, were positively correlated with Aβ pathology and negatively correlated with nesting scores. In contrast, the abundance of *Butyricicoccus* (highlighted in orange) that decreased in APP/PS1 control mice, was positively correlated with nesting scores and negatively correlated with Aβ pathology. Measures of Aβ pathology include the area of Aβ plaques, number of Aβ plaques, area of large Aβ plaques (>500 μm^2^), and area of small Aβ plaques (<500 μm^2^). n = 4 per group. * *p* < 0.05; ** *p* < 0.01; *** *p* < 0.001.

	Nesting		Area of Aβ Plaques		Number of Aβ Plaques		Aβ Plaques >500 μm^2^		Aβ Plaques <500 μm^2^	
	R	*p* Value	R	*p* Value	R	*p* Value	R	*p* Value	R	*p* Value
**Genera Abundance Increased in APP/PS1 Mice**										
** Acinetobacter**	−0.493	0.027 *	0.363	0.167	0.415	0.110	0.283	0.288	0.419	0.106
** Bacteroides**	−0.503	0.024 *	0.607	0.013 *	0.586	0.017 *	0.583	0.018 *	0.550	0.027 *
** Dubosiella**	−0.756	< 0.001 ***	0.543	0.030 *	0.649	0.007 **	0.331	0.210	0.680	0.004 **
** Eubacterium nodatum group**	−0.761	< 0.001 ***	0.505	0.046 *	0.500	0.049 *	0.411	0.114	0.488	0.055
** Family XIII AD3011 group**	−0.526	0.017 *	0.590	0.016 *	0.592	0.016 *	0.570	0.021 *	0.560	0.024 *
** Gemella**	−0.412	0.071	0.585	0.017 *	0.541	0.030 *	0.518	0.040 *	0.512	0.042 *
** Lachnospiraceae UCG 001**	−0.164	0.491	0.477	0.062	0.416	0.109	0.471	0.065	0.377	0.149
** Lactobacillus**	−0.064	0.789	0.278	0.316	0.313	0.238	0.181	0.502	0.323	0.221
** Marinomonas**	−0.626	0.003 **	0.598	0.014 *	0.647	0.007 **	0.463	0.071	0.649	0.007 **
** Megasphaera**	−0.425	0.062	0.339	0.199	0.415	0.110	0.263	0.325	0.424	0.102
** Rikenellaceae RC9 gut group**	−0.691	< 0.001 ***	0.592	0.030 *	0.502	0.048 *	0.536	0.032 *	0.462	0.072
** Vibrio**	−0.363	0.116	0.391	0.134	0.472	0.065	0.264	0.324	0.490	0.054
**Genera abundance decreased in APP/PS1 mice**										
** Alloprevotella**	0.320	0.169	−0.080	0.769	−0.154	0.569	−0.060	0.825	−0.168	0.535
** Butyricicoccus**	0.622	0.003 **	−0.509	0.044 *	−0.546	0.029 *	−0.433	0.094	−0.536	0.032 *
** Lachnospira**	0.150	0.528	−0.241	0.369	−0.121	0.656	−0.327	0.217	−0.066	0.808
** Ruminococcaceae UCG 010**	−0.163	0.493	0.381	0.145	0.398	0.127	0.343	0.193	0.383	0.142

## Data Availability

Data of the current study are available from the corresponding author on request.
